# Seeing is believing: believing in a just world reduces perceived inequality

**DOI:** 10.3389/fpsyg.2025.1531682

**Published:** 2025-07-31

**Authors:** Melvin John, Herbert Bless

**Affiliations:** Chair of Social Psychology, Department of Psychology, University of Mannheim, Mannheim, Germany

**Keywords:** perceived inequality, belief in a just world, motivated reasoning, subjective inequality, meritocratic beliefs

## Abstract

**Introduction:**

Inequality is one of the most pressing social issues of our time, yet individuals often differ in how they perceive and evaluate it. These subjective differences hinder the formation of a common understanding, making it difficult to reach consensus on how to address inequality. This research investigates the role of fairness beliefs-specifically just world beliefs (BJW) and meritocracy-in shaping individual perceptions of inequality.

**Methods:**

We conducted three studies across four independent samples (Ns between 543 and 36,281), using experimental, survey, and cross-national designs. Study 1 experimentally manipulated fairness beliefs. Study 2 analyzed two representative German samples. Study 3 employed multilevel modeling with data from 40 countries.

**Results:**

Fairness beliefs, particularly belief in a just world (BJW), consistently influenced both the perceived and evaluated size of financial inequality across all three studies. Experimental evidence (Study 1) showed a causal effect of BJW on both these components. In large-scale observational data (Studies 2 and 3), BJW remained a significant predictor of perceived and evaluated inequality across diverse national samples.

**Discussion:**

These findings suggest that BJW not only influences how people evaluate inequality but also shapes their perception of its scale. By identifying fairness beliefs as a core factor behind subjective inequality, this work provides insights into the psychological roots of disagreement about inequality and offers a foundation for addressing these divides in public discourse and policy.

## 1 Introduction

Financial inequality has arguably become one of the most important social issues of the 21st century. Barack Obama referred to financial inequality as a test for democracy that “will determine our future” (Obama, 2017), while Pope Francis called it a “sickness” that would tear apart the fabric of society (Brockhaus, 2020). Indeed, a large body of empirical research has linked financial inequality to many social ills (Wilkinson and Pickett, 2009), such as higher crime rates (Hsieh and Pugh, 1993), social conflict (Gimpelson and Treisman, 2018), and worse overall health (Pickett and Wilkinson, 2015; Sommet et al., 2018), possibly leading to an estimated 1.5 million premature deaths in OECD countries alone (Kondo et al., 2009). Unsurprisingly, financial inequality has become a frequent topic in public discourse and policy makers have begun to consider it as critical input to guide their economic and social policy decisions (Hauser and Norton, 2017).

It seems that most political discussions and scientific research on the negative consequences of financial inequality focus primarily on objective financial inequality, for example assessed by the Gini index. However, research in recent years has documented the crucial importance of how people perceive financial inequality. For example, preferences for redistribution (Bobzien, 2020; Niehues, 2014), emergence of social conflict (Gimpelson and Treisman, 2018), and subjective wellbeing (Bagherianziarat and Hamplova, 2022; Schalembier, 2019) are at least as strongly influenced by perceived as by objective inequality (for an overview of the effects of perceived inequality see Willis et al., 2022).

The pronounced influence of perceived financial inequality raises the question of what factors determine individuals' perception of inequality. Addressing this question, the present paper examines the potential role of individuals' belief in a just world (Lerner, 1980). In the following we first address the concept of perceived financial inequality, thereby differentiating between a more evaluative component (e.g., “I believe financial inequality is too large”) vs. the subjective perception of the size of financial inequality. We then elaborate on the concept of belief in a just world (BJW) and subsequently discuss potential influences of BJW on perceived financial inequality. We report three studies that examine this question in a preregistered experimental study (Study 1), a correlational study based on a representative national survey (Study 2), and a correlational multi-national study (Study 3).

When talking about financial inequality politicians and the media typically refer to actual levels of inequality and do not consider how people perceive inequality or what impact this perception might have (Hauser and Norton, 2017). However, research in recent years has shown that individual policy preferences as well as social ills that are typically linked to inequality depend less on the actual levels of inequality. Instead, how people subjectively perceive inequality is also of critical importance (e.g., Bobzien, 2020; Castillo et al., 2021; Gimpelson and Treisman, 2018; Oshio and Urakawa, 2014; Schneider, 2012; Willis et al., 2022).

Subjective inequality can in large parts be independent of “objective” inequality (García-Castro et al., 2022). Depending on who is asked, the actual extent of inequality is either systematically over- or underestimated (Gimpelson and Treisman, 2018; Hauser and Norton, 2017; Kiatpongsan and Norton, 2014; Kteily et al., 2017; Niehues, 2014; Norton and Ariely, 2011). Likewise, people struggle to correctly identify changes in inequality over time (Gimpelson and Treisman, 2018).

When investigating the consequences of subjective inequality past research has considered different components of subjective inequality (García-Castro et al., 2022). On the one hand, research has examined normative and evaluative aspects of financial inequality—for example, by asking respondents whether they think existing income inequality is too large (e.g., Bavetta et al., 2019; Castillo et al., 2021). Relatedly, Schmalor and Heine (2021) have developed a scale to measure the interpretation and evaluation of inequality. On the other hand, research has attempted to assess the perceived size of financial inequality. For example, individuals are asked to report income gaps, i.e., individuals estimate the income of specifically described high vs. low status persons (Castillo et al., 2021; Gimpelson and Treisman, 2018; Schneider, 2012). Moreover, to assess perceived size of financial inequality, other research has provided respondents with graphical displays of inequality and respondents then select the one that best represents the actual distribution (Gimpelson and Treisman, 2018; Niehues, 2014; Rodriguez-Bailon et al., 2017). Importantly, evaluations and perceived size of financial inequality capture different aspects and need not overlap. For example, individuals may perceive high levels of inequality but may evaluate inequality as positive or legitimate due to their beliefs about the origins or the consequences of inequality (García-Sánchez et al., 2022). Interestingly, the correlation between the different conceptualizations and operationalizations of subjective inequality can be rather weak, suggesting that different concepts are indeed captured by the different approaches (for a broader discussion on conceptualizations of financial inequality, see Jachimowicz et al., 2022).

Unlike “objective” financial inequality captured by the Gini Index (or comparable operationalizations, see De Maio, 2007), all variants of subjective inequality reflect substantial differences between individuals that share the same objective inequality situation. This raises the question of which factors shape the subjective perspective of inequality. In this respect prior research has, for example, demonstrated that perceptions of inequality are influenced by a persons' social environment (Cruces et al., 2013), political ideology (Kteily et al., 2017; Waldfogel et al., 2021), and by the media coverage of inequality (Diermeier et al., 2017). The present research similarly investigates potential causes of different subjective perspectives on financial inequality by focusing on individuals' beliefs in a just world (BJW; Lerner, 1980). In the next sections we review prior research on BJW and link it to subjective inequality.

The concept of BJW is based on the just world hypothesis by Lerner (1980), and represents a belief in a world where people generally get what they deserve. According to the just world hypothesis, having such a belief allows individuals to see their physical and social environment as orderly and predictable which reduces feelings of uncertainty and risk. A strong BJW may thus function as a psychological coping mechanism that protects against stress and anxiety in an uncertain world (Furnham, 2003). In other words, people for whom BJW serves this function actually *need* to belief the world is just and are therefore motivated to perceive reality in a way that confirms or at least does not contradict this belief (Hafer and Bègue, 2005).

Prior research has demonstrated that such motivations often lead people to perceive, interpret, and evaluate information in ways that align with their preexisting beliefs (e.g., Ditto and Lopez, 1992; Dunning et al., 1991; Kunda, 1990). In line with this perspective, people who have a strong BJW have been found to avoid or distance themselves from information that suggest the world to be unjust (Hafer, 2000; Pancer, 1988) and go so far as to derogate or even blame victims of crime for their own misfortune, because an innocent victim would contradict the view of the world as a just place, whereas a non-innocent victim does not (De Judicibus and McCabe, 2001; Montada, 1998). Applying this perspective to the perception of inequality, we suggest that people with a stronger BJW are motivated to evaluate inequality more positively and will perceive it to be lower to protect their BJW.

At first glance, this suggestion appears to overlap with considerations from system justification theory, which holds that people are motivated to legitimize and defend existing social arrangements—including inequality—due to a belief in meritocracy and a preference for hierarchical order (Jost et al., 2004; Napier and Jost, 2008). However, while both system justification theory and BJW address psychological mechanisms that support inequality, they differ in important ways. System justification theory describes a motivation to defend the status quo, often by rationalizing existing inequalities as fair or necessary. In contrast, BJW reflects a broader epistemic need to see the world as a just and orderly place (Furnham, 2003), which can lead individuals to cognitively downplay or deny the presence of injustice to maintain that belief. As a result, although both theories predict a tendency to justify inequality, their implications for perceived inequality diverge. Specifically, BJW is likely to be associated with *lower* perceived inequality, because acknowledging inequality would threaten the belief that the world is fair. By contrast, individuals high in system justification may *acknowledge* inequality but still evaluate it positively—seeing it as legitimate or deserved. Therefore, we would expect different patterns of results depending on whether BJW or system justification is used to predict perceptions of inequality: BJW may reduce perceived inequality, whereas system justification may not affect perceived levels directly, but rather shape how inequality is interpreted or justified.

To our knowledge no prior research has examined how BJW affects the perceived size of inequality. Indirect support for the potential role of BJW can be derived from research addressing constructs related to BJW. For example, Kteily et al. (2017) found that, due to motivated processing, individuals with a stronger social dominance orientation perceive lower racial or gender inequality, which was corroborated by Waldfogel et al. (2021), who demonstrated that motivational processes associated with social dominance orientation reduce individual vigilance to cues about inequality. Similarly, other research has found that compared to political conservatives, liberals perceive higher income and wealth inequality (Norton and Ariely, 2011) and perceive lower levels of economic mobility (Chambers et al., 2015; Davidai and Gilovich, 2015, 2018). More directly related to the present investigation, García-Sánchez et al. (2022) recently provided evidence that BJW influences individuals' perceived fairness and policy preferences regarding inequality, such that people with a weaker BJW deemed inequality less legitimate and redistributive policies more necessary. Furthermore, Kraus et al. (2017) showed that people in the USA with stronger just world beliefs estimate racial inequality to be lower.

While prior research suggests a relationship between Belief in a Just World (BJW) and subjective inequality, several open questions remain, and the present research aims to address these.

First, although related constructs such as social dominance orientation and political ideology have been shown to predict subjective financial inequality (e.g., social dominance orientation, see Kteily et al., 2017; Waldfogel et al., 2021; political ideology, see Norton and Ariely, 2011), the role of BJW specifically in shaping perceptions of financial inequality remains underexplored. Prior work that directly assessed BJW has focused on related but distinct outcomes, such as the perceived legitimacy of inequality (García-Sánchez et al., 2022) or racial inequality (Kraus et al., 2017). While studies such as Batruch et al. (2023) have examined the legitimizing role of school meritocracy and inequality, they did not directly examine perceptions of financial inequality as an outcome. The current research therefore seeks to extend this work by directly linking BJW to subjective financial inequality, encompassing both *perceived* and *evaluated* components (Study 1–3).

Second, much prior work on subjective inequality and its psychological antecedents has been conducted in U.S. samples (e.g., Davidai and Gilovich, 2015; Kteily et al., 2017; Norton and Ariely, 2011; Waldfogel et al., 2021) which raises questions about cross-cultural generalizability. Although more recent work has begun to expand beyond this context (Batruch et al., 2023; Castillo et al., 2019; Dekkers et al., 2022), the need remains for broader, multi-country data. We address this by drawing on representative survey data from Germany (Study 2) and a cross-national sample spanning 40 countries (Study 3) to assess the generalizability of the BJW–inequality relationship.

Third, to our knowledge past research on predictors of perceived financial inequality has not yet provided experimental evidence that allows for causal conclusions about the relationship. Relying only on correlational data cannot rule out the possibility that it is not different levels of BJW that lead to different perceptions of inequality, but that different perceptions of inequality may lead to different levels of BJW. We address this aspect in a preregistered experimental study (Study 1).

Fourth, past research has investigated subjective financial inequality by addressing an evaluative component or the perceived size of inequality (see e.g., García-Castro et al., 2019 for a discussion). Interestingly, some of the applied measures may confound these two aspects (e.g., measuring perceived inequality by asking respondents whether they believe existing levels of inequality are too large, e.g., Bavetta et al., 2019). Given the present focus on BJW it seems important to examine whether BJW not only influences the *evaluated* size of inequality but also whether the motivation to defend BJW already influences the input of this evaluation by affecting the *perceived* size of inequality. In other words, people with a stronger BJW may perceive lower levels of inequality irrespective of how fair they think inequality is. To our knowledge this possibility has not been considered in past research. Relatedly, we investigated the mechanism that links BJW to different measures of subjective inequality to assess whether the perceived size of inequality is connected to BJW via a different mechanism than the evaluated size of inequality (Study 2). Since the evaluated size of inequality likely captures unfairness beliefs about inequality too, whereas the mere perceived size of inequality does not, we expected that BJW affects the perceived size of inequality irrespective of unfairness beliefs about inequality, whereas we expected the effect of BJW on the evaluated size of inequality to be (partially) mediated by unfairness beliefs about inequality.

Based on the above considerations, the present research provides a unique combination of different methodologies (large scale survey data and experimental data) to comprehensively assess the effect of BJW on different measures of subjective inequality. We predicted a negative relationship between the strength of individual BJW and the perceived size of financial inequality. We examined this prediction in three studies across 4 separate samples (*N*s between 543 and 36,281).

All materials (e.g., analysis code, non-public data, and questionnaires) and an overview of all the used variables have been made publicly available on the OSF (https://osf.io/4ap63/?view_only=a807414c00524dbb9ebef1e50a7946b1) and all studies, measures, manipulations and data exclusions are reported. Preregistrations for Study 1 (https://aspredicted.org/KYB_W9V) and Study 2b (https://aspredicted.org/V38_1GY) can be found on www.aspredicted.org. Unless otherwise stated, data were analyzed using R, version 4.2.1 (R Core Team, 2021).

## 2 Study 1

The goal of Study 1 was to provide first evidence for a causal effect of BJW on the perceived size of inequality by assessing the effects of BJW on perceived inequality experimentally. We build on earlier research that has shown that fairness beliefs can be momentarily manipulated by providing participants information about the societal (un-)fairness in the country they live in (Laurin et al., 2011). We expected that participants who were told that the country they live in is relatively fair would report higher BJW and subsequently report to perceive less inequality than participants who were told that the country they live in is relatively unfair.

With the focus of Study 1 on the causal effect of BJW, we further addressed the disentangling of the perceived size and the evaluation component of subjective inequality.

### 2.1 Method

The design, procedure, and analyses of this study were preregistered (https://aspredicted.org/KYB_W9V).

#### 2.1.1 Data

We collected data from 600 German participants (42% female, 57% male, 1% non-binary; *M*_age_ = 29, *SD*_age_ = 9) via the online recruiting platform Prolific (www.prolific.co). Participants were compensated with £0.80 (£8 per hour). The sample size was pre-determined with an a-priori power-analysis. By collecting data from 600 participants, we achieved 95% power to detect a small effect size (*d* ≈ 0.3), given a type I error rate of 5%. We excluded a total of 57 participants who either failed a comprehension check or did not follow the instructions related to the manipulation (described in more detail below). Although the study was preregistered, these specific exclusion criteria had not been anticipated beforehand. Some participants provided examples that did not correspond to their assigned condition—for instance, describing fairness while being in the unfairness condition or vice versa—while others gave unrelated or evaluative responses (e.g., expressing general agreement or disagreement with the fairness index). These participants were excluded to ensure that only those who engaged with the manipulation as intended were included in the analysis. Therefore, the final sample size comprised 543 German participants (43% female, 57% male, 1% non-binary; *M*_age_ = 29, *SD*_age_ = 9). The 57 excluded participants were about equally distributed across the two experimental conditions (24 vs. 33).

#### 2.1.2 Materials

##### 2.1.2.1 Manipulation of belief in a just world

To manipulate BJW we adapted a paradigm first introduced by Laurin et al. (2011). Participants were randomly assigned to one of two between-subjects conditions in which they were asked to read a text about new findings from sociology that supposedly allow to measure the fairness of society with a so-called fairness-index. This fairness-index would for instance indicate how much individual success is determined by individual effort and hard work rather than external circumstances such as race, gender, or unfair advantages. In the low BJW condition participants were told that according to this index Germany would be a relatively unfair country, i.e., individual success would mostly be determined by external circumstances. In the high BJW condition participants were analogously told that according to this index Germany would be a relatively fair country, i.e., individual success would be determined by individual efforts and hard work. In a second step participants were then required to provide an example illustrating that the German society is either relatively fair (high BJW condition) or unfair (low BJW condition). We assessed the success of the manipulation with a German 6-item version of the Global Belief in a Just World scale (Dalbert et al., 2002).

Participants were fully debriefed after the study. They were informed that the Fairness Index described in the experiment was fictitious and created for research purposes. The true aim of the study—to examine the impact of belief in a just world on perceptions of inequality—was explained.

##### 2.1.2.2 Perceived size of inequality

To assess the perceived size of financial inequality independently of an evaluative component, we used a series of six bar charts, each with 5 bars, depicting different income distributions between income-quintiles in Germany. Respondents were asked to choose the bar chart that they thought depicted the distribution of income in Germany (see e.g., Rodriguez-Bailon et al., 2017 for a similar measure). The bar charts were arranged in ascending order from left to right according to the degree of inequality they displayed. Starting from a Gini index of 22 on the far left, the Gini index increased by 5 for each bar chart further to the right, ending with a Gini index of 47 on the far right. Thereby the actual distribution of income between the quintiles in Germany at the time was depicted in the third bar chart from the left (Gini index of 32). We provided participants with an in-depth explanation and examples of the quintile bar-charts beforehand and excluded participants from subsequent analyses who indicated that they did not understand the charts completely.^1^

##### 2.1.2.3 Evaluated size of inequality

We relied on a German version of the 4-item subscale for subjective inequality of the Subjective Inequality Scale (SIS) by Schmalor and Heine to “assesses how people interpret and evaluate the level of inequality around them” (Schmalor and Heine, 2021, p. 2). In contrast to more concrete measures—such as those that ask respondents to select from visual representations like bar charts depicting different distributions of income, as described above—the SIS captures general impressions and evaluative judgments rather than precise estimations of economic data. Participants were asked how much statements such as “Almost all the money that is earned goes to only a few people” and “Real opportunities to succeed in life are only available to the wealthy” apply to describe inequality in Germany, using a Likert scale ranging from 1 (does not apply at all) to 7 (applies completely). We computed a summary score across the 4 items (*M* = 4.46, SD = 1.18, α = 0.78).

### 2.2 Results and discussion

Reflecting a successful manipulation of BJW, participants in the low BJW condition reported lower BJW (*M* = 2.48, *SD* = 0.97) than participants in the high BJW condition (*M* = 2.76, *SD* = 0.99), *t*_(541)_ = 3.38, *p* < 0.001, *d* = 0.29, *95%CI* [0.12, 0.46].

**Figure 1:**
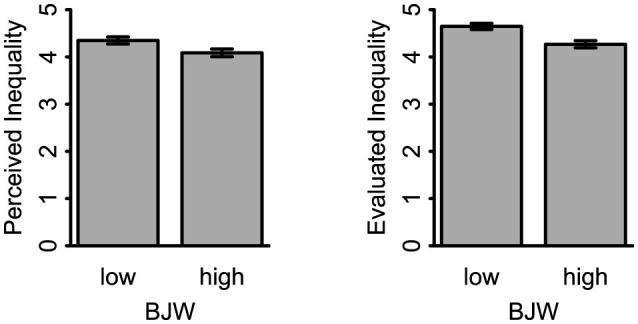
Effect of belief in a just world on the perceived and on the evaluated size of income inequality. The left panel shows the effect on the perceived size of income inequality; the right panel shows the effect on the evaluated size of income inequality. Error bars show 95% confidence intervals. low, low BJW condition; high, high BJW condition.

In a second step, we tested the effects of BJW on subjective inequality (with a one-sided *t*-test, as preregistered). Consistent with our hypothesis we found that participants in the high BJW condition perceived the size of inequality to be lower (*M* = 4.09, *SD* = 1.33) than participants in the low BJW condition (*M* = 4.35, *SD* = 1.25), *t*_(541)_ = −2.37, *p* = 0.009, *d* = −0.20, *95%CI* [−0.37, −0.03]. Moreover, participants in the high BJW condition evaluated inequality more positively (*M* = 4.65, *SD* = 1.08), than participants in the low BJW condition (*M* = 4.27, *SD* = 1.25), *t*_(541)_ = −3.78, *p* < 0.001, *d* = −0.33, *95%CI* [−0.49, −0.16] (see Figure 1).^2^

In combination the observed results provide first support for the hypothesis that changes in BJW cause changes in subjective financial inequality. By addressing the size and the evaluations component separately, the results indicate that this causal effect pertains to both aspects of financial inequality. However, it is worth noting that the effect on perceived inequality was less robust when using the full sample without applying additional (non-preregistered) exclusions, whereas the effect on evaluated inequality remained stable. This suggests that the effect on perceived inequality may be somewhat more sensitive to sample composition.

## 3 Study 2

In Study 1 we assessed the effect of BJW on perceived inequality experimentally, to allow for causal conclusions. Study 2 was designed to complementarily assess the generalization of the association between BJW and the perception of financial inequality beyond the experimental context. We conducted Study 2 twice (Study 2a and 2b) with non-overlapping representative samples which (a) yields externally more valid effect estimates but also (b) allows to assess the robustness of our effects in Study 2a via direct replication in Study 2b. We pre-registered our analyses for Study 2b on www.aspredicted.org (https://aspredicted.org/V38_1GY).

Building on the results from Study 1, we again considered different components of subjective inequality (García-Castro et al., 2022; Schmalor and Heine, 2021) by assessing perceived size of inequality and evaluations of inequality. We expected that participants with a strong BJW perceive the size of inequality to be lower and evaluate inequality less negatively than participants with a weaker BJW. Moreover, we exploratively investigated whether the effect of BJW on the evaluated but not on the perceived size of inequality is mediated via a different pathway. Specifically, the evaluation of inequality is strongly influenced by general unfairness beliefs about inequality (see e.g., Schmalor and Heine, 2021), whereas the perceived size of inequality is not (see e.g., John et al., 2025). We thus expected that the effect of BJW on evaluations of inequality but not on the perceived size of inequality is mediated by these unfairness beliefs about inequality.

### 3.1 Method

#### 3.1.1 Data

We relied on an online panel from Bilendi and Respondi to collect data from 1096 respondents for Study 2a and 1102 respondents for our replication, Study 2b. We used quotas for gender, age, income, federal state, occupation status, and education, such that the distribution on these variables reflected their distribution in the German population. As preregistered, we excluded participants who failed a comprehension check. The final sample size comprised 839 respondents for Study 2a (49% female, *M*_age_ = 47, *SD*_age_ = 17) and 743 respondents for Study 2b (48% female, *M*_age_ = 45, *SD*_age_ = 14).^3^

#### 3.1.2 Measures

Following we do not report all variables that were included in the questionnaires but focus on and report only the variables directly relevant to the research question at hand. An overview of the focal variables and intercorrelations is displayed in Table 1.

**Table 1:** Descriptive statistics of focal variables and covariates.

**Variable**	***M***	***SD***	**1**	**2**	**3**	**4**
**Study 2a**						
1. Inequality: perceived size	37.02	8.03	–			
2. Inequality: evaluation	4.82	1.22	0.17^***^	0.80		
3. Inequality: unfairness beliefs	5.74	1.18	0.06^†^	0.46^***^	0.84	
4. BJW	2.92	1.21	−0.11^***^	−0.15^***^	−0.33^***^	0.85
**Study 2b**						
1. Inequality: perceived size	37.35	8.18	–			
2. Inequality: evaluation	5.93	1.63	0.19^***^	0.80		
3. Inequality: unfairness beliefs	7.03	1.69	0.05	0.43^***^	0.85	
4. BJW	3.47	1.59	−0.07^*^	−0.14^***^	−0.35^***^	0.87

Study 2a N = 839. Study 2b N = 743. Values in main diagonal of correlations show internal consistency reliability. BJW, Belief in a just world. In Study 2a scales for variables 2–4 ranged from 1 to 7. In Study 2b scales for variables 2–4 ranged from 1 to 9.

† *p* < 0.1.

* *p* < 0.05.

*** *p* < 0.001.

##### 3.1.2.1 Belief in a just world

We measured BJW using a German 6-item version of the Global Belief in a Just World scale (Dalbert et al., 2002). Participants indicated their agreement on a scale from 1 (*disagree completely*) to 7 (*agree completely*) in Study 2a and from 1 (*disagree completely*) to 9 (*agree completely*) in Study 2b to statements such as “I think that the world is generally fair”.^4^ We computed a summary score across all 6 items to use in the analyses (Study 2a: *M* = 2.92, *SD* = 1.21, α = 0.85; Study 2b: *M* = 3.47, *SD* = 1.59, α = 0.87).

##### 3.1.2.2 Perceived size of inequality

We measured the perceived size of inequality as we did in Study 1, asking respondents to choose between 6 different quintile bar charts which corresponded to Gini-values between 22 and 47 (Study 2a: *M* = 37.02, *SD* = 8.03; Study 2b: *M* = 37.35, *SD* = 8.18). As in Study 1 we provided participants with a lengthy explanation and examples of the quintile bar-charts beforehand and excluded participants from subsequent analyses who indicated that they did not understand the charts completely. In addition to this comprehension check, Study 2b included an comprehension check in which participants were shown four bar charts—each depicting a different distribution of income across five quintiles—and were asked to identify the chart that showed the highest level of inequality. We excluded participants from Study 2b who failed this comprehension check. We made an English version of the explanation and question publicly available on the OSF (here). An English version of the comprehension check question from Study 2b is also publicly available on the OSF (here).

##### 3.1.2.3 Evaluated size of inequality

As in Study 1, we relied on the first 4-item subscale for subjective inequality from the SIS (Schmalor and Heine, 2021). In Study 2a we used a scale from 1 (*does not apply at all*) to 7 (*applies completely*) and in Study 2b we used a scale from 1 (*does not apply at all*) to 9 (*applies completely*). We computed a summary score to use in the analyses (Study 2a: *M* = 4.82, *SD* = 1.22, α = 0.80; Study 2b: *M* = 5.93, *SD* = 1.63, α = 0.80).

##### 3.1.2.4 Unfairness beliefs about inequality

We relied on the second 4-item subscale for unfairness beliefs about inequality from the SIS (Schmalor and Heine, 2021). In Study 2a we used a scale from 1 (*does not apply at all*) to 7 (*applies completely*) and in Study 2b we used a scale from 1 (*does not apply at all*) to 9 (*applies completely*). We computed a summary score to use in the analyses (Study 2a: *M* = 5.74, *SD* = 1.18, α = 0.84; Study 2b: *M* = 7.03, *SD* = 1.69, α = 0.85).

##### 3.1.2.5 Covariates

We controlled for respondents age, income, gender (0 = *male*), and education (*low, medium, high*), because these variables have been associated with our focal variables in previous research (see e.g., Schneider, 2012).

### 3.2 Results and discussion

In a first step we looked at the bivariate correlation of our two measures of perceived inequality (see Table 1) for an overview of the focal variables and intercorrelations. The two measures correlated positively (Study 2a: *r* = 0.18, *p* < 0.001; Study 2b: *r* = 0.19, *p* < 0.001), though the correlation was not very strong. This further indicates that both measures capture distinguishable aspects of perceived inequality—the graphic measure specifically captures perceived size of income inequality, whereas the SIS more likely captures evaluations of inequality. This assumption is reinforced by the correlations of these two measures with unfairness beliefs about inequality. Only the evaluation (Study 2a: *r* = 0.46, *p* < 0.001; Study 2b: *r* = 0.43, *p* < 0.001) but not the perceived size of inequality (Study 2a: *r* = 0.06, *p* = 0.054; Study 2b: *r* = 0.05, *p* = 0.135) correlated significantly with these unfairness beliefs. We explored the implications of this difference in more detail below.

In a second step we used multiple regression analysis to test our hypothesis that a stronger BJW leads to a lower perceived size and more positive evaluation of inequality. Since we had two different measures of subjective inequality in each study, we conducted the analysis twice for each study, once with each measure of subjective inequality as the dependent variable.

As can be seen in Table 2, participants with a stronger BJW reported less subjective inequality. This effect was found in both Study 2a and 2b, both for the measure using the bar charts that specifically captured the perceived size of income inequality (Study 2a: *b* = −0.12, *p* < 0.001, $\eta_{\text{p}}^{2}$ = 0.015; Study 2b: *b* = −0.12, *p* = 0.001, $\eta_{\text{p}}^{2}$ = 0.014) and for the measure using the subjective inequality subscale of the SIS which captures evaluations of inequality (Study 2a: *b* = −0.13, *p* < 0.001, $\eta_{\text{p}}^{2}$ = 0.016; Study 2b: *b* = −0.11, *p* = 0.003, $\eta_{\text{p}}^{2}$ = 0.012). The effects of BJW on both measures of subjective inequality were similar in size and larger than effect sizes that are typically observed in social psychological research (see e.g., Entringer et al., 2021 for a discussion on effect sizes in psychological research). Furthermore, the effects were quite robust and replicated almost exactly in Study 2b.^5^

**Table 2:** Results from linear regressions predicting different measures of subjective inequality.

**Variable**	**Inequality: perceived size**		**Inequality: evaluation**	
	***b***	***95% CI***	***b***	***95% CI***
**Study 2a**				
Intercept	0.19^*^	[0.00, 0.38]	0.43^***^	[0.25, 0.62]
Age	0.08^*^	[0.01, 0.15]	0.10^**^	[0.03, 0.17]
Income	−0.02	[−0.06, 0.03]	−0.07^**^	[−0.11, −0.02]
Gender: female	−0.38^***^	[−0.51, −0.24]	−0.14^*^	[−0.28, −0.01]
Education: med.	−0.00	[−0.16, 0.15]	−0.18^*^	[−0.33, −0.03]
Education: high	0.26^*^	[0.05, 0.46]	−0.34^**^	[−0.54, −0.14]
BJW	−0.12^***^	[−0.19, −0.05]	−0.13^***^	[−0.19, −0.06]
**Study 2b**				
Intercept	0.15	[−0.02, 0.33]	0.15	[−0.02, 0.33]
Age	−0.06	[−0.14, 0.02]	0.07	[−0.00, 0.15]
Income	0.01	[−0.06, 0.09]	−0.14^***^	[−0.21, −0.07]
Gender: female	−0.39^***^	[−0.53, −0.24]	−0.05	[−0.19, 0.09]
Education: med.	−0.09	[−0.29, 0.11]	−0.16	[−0.36, 0.05]
Education: high	0.15	[−0.05, 0.35]	−0.18	[−0.38, 0.02]
BJW	−0.12^**^	[−0.19, −0.05]	−0.11^**^	[−0.19, −0.04]

Study 2a N = 835. Study 2b N = 743. BJW, Belief in a just world (higher values indicate stronger belief).

* *p* < 0.05.

** *p* < 0.01.

*** *p* < 0.001.

**Figure 2:**
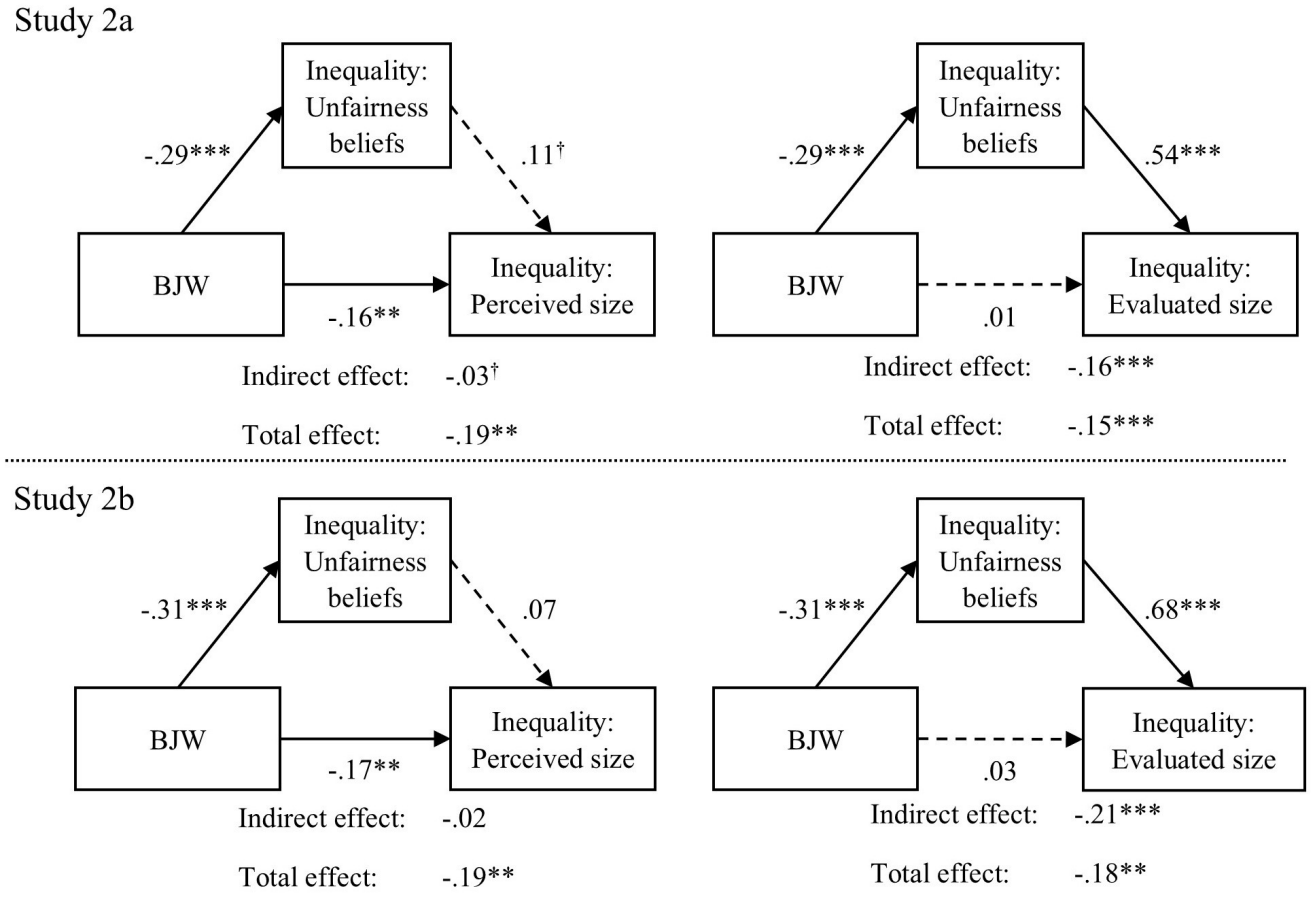
Results from mediation analyses, predicting subjective inequality from bjw via unfairness beliefs about inequality. Dashed lines show insignificant effects. BJW, Belief in a just world. Estimates are standardized coefficients. The two panels on the top display the results for Study 2a; the two panels on the bottom display the results for Study 2b; the two panels on the left display the results with the perceived size of inequality as the dependent variable; the two panels on the right display the results with the evaluated size of inequality as the dependent variable. We included participant age, income, gender, and education as control variables. For all models RMSEA = 0; CFI = 1 and TLI = 1. ^†^*p* < 0.1. ^**^p < 0.01. ^***^p < 0.001.

In a third step we investigated whether BJW affects the two different measures of subjective inequality via different pathways. Indeed, as can be seen in Figure 2 we found that the effect of BJW on the evaluated size of inequality is completely mediated by unfairness beliefs about inequality, whereas the effect of BJW on the perceived size of inequality is not. Therefore, not only do these results reinforce the notion that perceived size and evaluation of inequality need to be considered separately but, crucially, they also suggest that the effect of BJW on perceived size of inequality is completely independent from general unfairness beliefs about inequality. One may speculate that BJW affects the perceived size of inequality via more upstream processes that guide individual attention and sensitivity toward inequality but that are themselves unrelated to unfairness beliefs about inequality.

Overall, the results point to BJW as an important predictor of subjective inequality that may explain the interindividual differences in inequality perception that were observed in prior research (e.g., Hauser and Norton, 2017; Norton and Ariely, 2011). Based on representative samples, the results from Study 2a and 2b attest to the generalizability of the results beyond the experimental context of Study 1 and to the robustness of the effects.

## 4 Study 3

So far, Study 1 demonstrates that BJW causally predicts perceived size and evaluations of inequality. Furthermore, Study 2 complements these findings with correlational data from the use of a representative sample and thus provides first evidence for generalizability. However, both studies are based on German samples and may therefore not generalize to other countries. To address this issue, in Study 3 we tested the generalizability of our results by relying on large scale international survey data. Furthermore, we explored whether economic and cultural country characteristics moderate the effect of individual BJW on the perceived size and evaluations of financial inequality. As an economic characteristic we considered the objective degree of income inequality as a moderator because it may be more difficult to intentionally avoid cues about inequality the higher objective inequality is. As a cultural characteristic, we considered the degree of religiosity, which has been shown to serve a palliative psychological function similar to BJW by helping individuals cope with societal stressors and perceived injustice (see e.g., Ano and Vasconcelles, 2005; Gebauer and Sedikides, 2021). Religiosity can offer meaning, perceived moral order, and assurances of ultimate justice—often through beliefs in divine justice or life-after-death compensation. These beliefs may reduce the psychological need to engage with structural inequalities in the present world. In line with system justification theory (Jost et al., 2004), religious beliefs can promote acceptance of societal hierarchies as legitimate or divinely ordained. Therefore, in highly religious contexts, the effect of individual BJW on perceptions and evaluations of inequality may be weaker because religiosity already fulfills a similar function—buffering individuals against the distress that might arise from recognizing inequality. Put differently, when religiosity is high, individuals may be less reliant on personal BJW to maintain a sense of justice in the world, thus attenuating its predictive power.

To the best of our knowledge there exists no international dataset that contains scores on a psychometrically validated BJW-scale and a measure for perceived or evaluated size of inequality. However, as argued in the just world hypothesis (Lerner, 1980), the essence of BJW is the belief that people get what they deserve (Furnham, 2003), which therefore is conceptually closely related to meritocratic beliefs about what it takes to be successful in this world—whether success can be earned through individual hard work and ambition, or whether it is due to unearned advantages such as parental wealth and social status. This perspective also parallels the manipulation we used in Study 1, where we successfully manipulated participants BJW by either providing meritocracy-confirming or -disconfirming information. In Study 3 we capitalized on this overlap by operationalizing BJW in terms of meritocratic beliefs. We expected that respondents with stronger meritocratic beliefs perceive less inequality and be less inclined to evaluate it as being too large. Furthermore, we expected that this effect would be stronger in countries with lower objective income inequality and lower religiosity.

### 4.1 Method

#### 4.1.1 Data

We relied on data from the International Social Survey Programme from the social inequality module from the year 2009 (ISSP; ISSP Research Group, 2017). The ISSP 2009 comprises 56,021 individuals from 41 countries with diverse cultural backgrounds (e.g., European, Asian, African, South American). Due to missing information on the focal variables, the final sample comprised 36,281 individuals (53% female, *M*_age_ = 46, *SD*_age_ = 16) from 40 countries.

#### 4.1.2 Measures

##### 4.1.2.1 Meritocratic beliefs

We relied on 8 different items from the ISSP survey that capture different meritocratic beliefs. Respondents were asked to indicate on a scale from 1 (*essential*) to 5 (*not important at all*) how essential different aspects were to get ahead in life. These aspects were parental wealth, individual ambition, and hard work, knowing the right people, having political connections, giving bribes, and a persons' race, and gender. We computed a summary score of these items such that higher values reflected stronger meritocratic beliefs (we reverse scored the items for individual ambition and hard work, *M* = 3.47, *SD* = 0.57, α = 0.67).

##### 4.1.2.2 Perceived inequality

Paralleling our approach in Study 2 we operationalized perceived inequality in two ways to capture the different aspects of perceived inequality—how large it is perceived, and how this size is evaluated.

To measure perceived size of inequality we used an item from the ISSP survey that is similar to our measure for perceived size of inequality from Study 2. Specifically, respondents were asked to choose between 5 different pyramid charts displaying different degrees of stratification the chart they thought represented the society they live in. Following the approach in earlier research (Bobzien, 2020; Gimpelson and Treisman, 2018; Niehues, 2014) we treated each bar in a chart as an income class and the area of a bar as representative of the population share in that income class (with each mm^2^ as an observation in that income class). We then calculated an unbiased Gini-index for each of the pyramid charts according to the following formula (Damgaard and Weiner, 2000; Dixon et al., 1987): $G={\frac{\overset{n}{\underset{i=1}{\sum }}\left(2i-n-1\right)y_{i}}{n\bar{y}}}^{*}\frac{n}{n-1}$, where *n* is the total area of all bars in a chart (in mm^2^), *y*_i_ is the income of the i'th observation, and $\bar{y}$ is the average income. The so-calculated Gini-indices of the five charts were 0.42 (A), 0.34 (B), 0.29 (C), and 0.20 (D and E). Due to its graphical nature, this measure is conceptually similar to our measure for perceived size from the first two studies.

To measure the evaluated size of inequality we used another item from the ISSP survey. Respondents were asked on a scale from 1 (*strongly agree*) to 5 (*strongly disagree*) to indicate their agreement with the statement that income differences in their country are too large. We reverse scored this item so that higher values indicate that inequality is evaluated as too large.

##### 4.1.2.3 Objective income inequality

We relied on the Gini index for income inequality from the World Bank as our measure for objective income inequality. Since the ISSP social inequality module was fielded in 2009 we also used Gini indices from the year 2009. Whenever a Gini index for a specific country was not available for the year 2009, we used the Gini index from the year closest to 2009 as a substitute.

##### 4.1.2.4 Religiosity

To operationalize country level religiosity, we relied on a question from the Gallup World Poll from 2005 to 2009. Respondents were asked the yes-no question whether religion is an important part of their daily life. We used the country level proportions of “yes”-answers published by Diener et al. (2011) as our measure for country level religiosity.

##### 4.1.2.5 Covariates

Paralleling our approach from Study 2, we controlled for respondents' gender (0 = male), age, education (in years of schooling) an income. To harmonize income data across countries in the ISSP 2009 dataset, we standardized the income variable within each country by converting it to a z-score. This ensured that income values represented individuals' relative positions within their national income distributions, adjusting for country-specific differences in income scales. The resulting standardized scores allow for meaningful cross-national comparisons based on relative income standing. An overview of the intercorrelations of the focal variables is displayed in Table 3.^6^

**Table 3:** Bivariate correlations of focal variables.

**Variable**	**1**	**2**	**3**	**4**
1. Perceived size of inequality	–	0.14^***^	−0.03^***^	–
2. Evaluation of inequality	0.67^***^	–	−0.03^***^	–
3. Meritocratic beliefs	−0.66^***^	−0.53^***^	–	–
4. Religiosity	0.40^*^	0.00	−0.27^†^	–
5. Objective income inequality (Gini)	0.32^*^	−0.10	−0.21	0.57^***^

N = 36,281 individuals in 40 countries. Values above the diagonal are correlations on the individual level (i.e., country-mean centered variables). Values below the diagonal are correlations on the country level (i.e., aggregated variables).

† *p* < 0.1.

* *p* < 0.05.

*** *p* < 0.001.

#### 4.1.3 Analysis strategy

In the ISSP data respondents are nested in countries. To account for this multilevel structure of the data, we used multilevel modeling. Following recommendations by Enders and Tofighi (2007), we centered all individual level, numeric predictor variables within countries and then standardized all numeric variables (including country level variables) between countries. Through this procedure the fixed effect coefficients from the multilevel model become standardized point estimates akin to standardized regression coefficient in OLS regression and can therefore be interpreted more easily. Moreover, this procedure is recommended when investigating cross-level interactions (Enders and Tofighi, 2007).

### 4.2 Results and discussion

As can be seen in Table 3 on the individual level the measure for perceived size of inequality and the measure for the evaluated size of inequality correlated positively but only weakly (*r* = 0.14, *p* < 0.001), suggesting that perceived and evaluated size of inequality indeed are related but separable constructs.

In a second step we tested our hypothesis that meritocratic beliefs are negatively related with both the perceived and evaluated size of inequality. Consistent with our hypothesis we found that people with stronger meritocratic beliefs perceived lower levels of inequality and were less inclined to evaluate inequality as too large (see Model 1 in Table 4).^7^

**Table 4:** Fixed effects from multilevel regression predicting perceived and evaluated size of inequality.

**Variable**	**Model 1**		**Model 2**	
	**zPE**	**95% CI**	**zPE**	**95% CI**
**Perceived size of inequality**				
Intercept	0.15	[−0.12, 0.43]	0.18	[−0.11, 0.46]
Religiosity (R)	0.17	[−0.04, 0.38]	0.19^†^	[−0.03, 0.41]
Objective income inequality (Gini)	−0.01	[−0.18, 0.17]	0.07	[−0.12, 0.25]
Meritocratic beliefs (MB)	−0.03^***^	[−0.05, −0.02]	−0.03^†^	[−0.05, 0.00]
MB × R			0.01	[−0.01, 0.03]
MB × Gini			0.02^*^	[0.00, 0.04]
**Evaluated size of inequality**				
Intercept	−0.04	[−0.27, 0.19]	0.00	[−0.24, 0.25]
Religiosity (R)	0.02	[−0.16, 0.19]	0.06	[−0.13, 0.25]
Objective income inequality (Gini)	−0.10	[−0.25, 0.04]	−0.06	[−0.22, 0.10]
Meritocratic beliefs (MB)	−0.04^*^	[−0.08, −0.01]	−0.01	[−0.07, 0.04]
MB × R			0.03	[−0.02, 0.07]
MB × Gini			0.03	[−0.01, 0.07]

*N* = 36,281 individuals from 40 countries. We controlled for age, gender, income, and education on the individual level. Religiosity and objective income inequality were measured on the country level.

† *p* < 0.1.

* *p* < 0.05.

*** *p* < 0.001.

In a third step we investigated whether the country level characteristics income inequality and religiosity moderate the effect of meritocratic beliefs on perceived and evaluated size of inequality. We found that the negative effect of meritocratic beliefs on the perceived size of inequality is stronger in countries with lower income inequality (see Model 2 in Table 4). However, we did not find that the country level characteristics moderated the effect of meritocratic beliefs on the evaluated size of inequality.^8^

Compared to the effect sizes in Study 2 the main effects of meritocratic beliefs on perceived and evaluated size of inequality were smaller in Study 3, which may suggest that the relationship between BJW and perceptions of inequality is stronger in Germany than in other countries. More likely however, the smaller effect sizes are due to lower reliabilities of the variables in Study 3. First, our measure for meritocratic beliefs in Study 3 had a comparatively low internal consistency reliability at α = 0.67, compared to the high internal consistency reliability of α = 0.85 of our measure for BJW in Study 2. Second, we explained our measure for perceived size of inequality and the bar charts extensively to respondents and probed for difficulties the respondents might have had with understanding this question in Study 1 and 2. In the ISSP survey we used in Study 3 the charts are not explained specifically to respondents and which likely led to higher measurement error of the perceived size of inequality in Study 3 (see also Knell and Stix, 2020 for potential problems with interpreting the respective figures). Similarly, the measurement error of our measure for the evaluation of inequality was likely higher in Study 3 because the ISSP data only contained a 1-item scale for the evaluation of inequality, whereas in Study 2 we relied on a 4-item scale. The effect sizes we found in Study 3 are therefore likely a conservative, lower bound estimate of the true effect sizes which thus are likely closer to the effects we found in Study 2.

Overall, the results of Study 3 show that the effects from Study 1 and 2 generalize beyond German samples. Generally, people who have a stronger BJW perceive inequality to be lower and evaluate it less negatively than people with a weaker BJW. Moreover, the results show that the strength of this effect may vary between countries depending on the actual level and cultural acceptance of inequality.

## 5 General discussion

The present investigation contributes to the growing literature on predictors of subjective inequality by examining the influence of Belief in a Just World (BJW) on both perceived and evaluated inequality. While prior research has examined psychological and ideological factors underlying inequality perceptions—such as political ideology, social dominance orientation, or media exposure (Cruces et al., 2013; Diermeier et al., 2017; García-Sánchez et al., 2022; Kraus et al., 2017; Kteily et al., 2017; Norton and Ariely, 2011; Waldfogel et al., 2021)—and others have explored the legitimizing function of meritocratic or just-world beliefs (Batruch et al., 2023; Dekkers et al., 2022), our work extends this literature in several important ways.

First, our studies provide causal evidence for the influence of BJW on subjective financial inequality, using an experimental manipulation (Study 1) to temporarily shift fairness beliefs—a method not employed in studies such as Dekkers et al. (2022), which rely on observational data. While previous research (e.g., Batruch et al., 2023) has shown that meritocratic beliefs may serve to justify inequality, our work focuses specifically on how these beliefs affect the *perceived size* of financial inequality, rather than attitudes or legitimacy judgments connected to inequality.

Second, considering the variety of measures for subjective inequality in prior research (for a discussion see e.g., García-Castro et al., 2019), we included measures for both the perceived and evaluated size of inequality in all three studies. We found that these measures were only weakly correlated on the individual level, corroborating the notion that subjective inequality is not a unidimensional construct but subsumes different, separable components (see e.g., García-Castro et al., 2022; Willis et al., 2022). Interestingly, we found that BJW affects the two components via different mechanisms (Study 2). The effect on the evaluated size of inequality was mediated by unfairness beliefs about inequality, whereas the effect on the perceived size of inequality was completely independent of these unfairness beliefs. Nevertheless, we found that BJW affects both components—the perceived and evaluated size of inequality—equally strongly. Combined, this result indicates that BJW not only motivates people to evaluate inequality differently but also influences the input of these evaluations by affecting the perceived size of inequality.

Third, unlike much prior research that draws primarily from U.S. samples (e.g., Davidai and Gilovich, 2015; Kteily et al., 2017; Norton and Ariely, 2011; Waldfogel et al., 2021), our studies include representative German samples and a global sample spanning 40 countries, offering initial insights into the (cross-cultural) generalizability of these effects. Although studies such as Batruch et al. (2023) and Castillo et al. (2019) have also included non-U.S. populations our work contributes by examining both perceived and evaluated inequality in diverse samples and also looked at potential country-level moderators of the effects (Study 3).

### 5.1. Caveats and future research

The results clearly support the hypothesis that BJW affects the perceived and evaluated size of inequality, yet some caveats remain which may stimulate further research on this topic. First, while the results overall show that the effect generalizes across a heterogeneous sample of 40 countries, the effect within countries is less consistent (see Supplementary Table S1 for country-wise effects). Though we indirectly addressed this issue through our moderator analysis, future research could shed more light on the exact nature of the relationship and identify further country-level moderators which may help explain why the effect is less pronounced in some countries.

Second, the results indicate that BJW is related to the perceived size of inequality via a different mechanism than to the evaluated size of inequality. While the impact of BJW on the evaluated size of inequality is mediated by unfairness beliefs regarding inequality, its effect on the perceived size of inequality, surprisingly, remains independent of these unfairness beliefs. This raises the question of what mechanism underlies the effect of BJW on the perceived size of inequality. One explanation may be that questions about the perceived size of inequality—which require respondents to accurately estimate inequality levels—are cognitively demanding (Hauser and Norton, 2017). As a result, rather than engaging in detailed assessments, individuals may rely on heuristic cues, such as their general belief that the world is fair, to generate a plausible answer. In other words, the impact of BJW on perceived inequality may not stem solely from motivated reasoning (i.e., defending a just worldview), but also from heuristic processing—where BJW serves as a cognitive shortcut (for a discussion on heuristic processing see e.g., Bohner et al., 1995).

Third, it is important to acknowledge that in Study 1, the effect of BJW on perceived inequality was no longer statistically significant when the full sample was analyzed without applying non-preregistered exclusion criteria. In contrast, the effect on evaluated inequality remained robust. This suggests that the observed causal effect on perceived inequality may be more sensitive to sample composition and may not be as stable as the effect on evaluations of inequality. Future research should further investigate the robustness of this finding and clarify under what conditions BJW reliably affects perceived inequality.

Understanding whether BJW influences perceptions of inequality through motivated or heuristic processing is practically relevant, as these two routes imply different points of intervention. If heuristic processing dominates, perceptions might be more malleable through changes in information presentation, framing, or education about actual inequality levels. In contrast, if motivated reasoning drives the effect, such perceptions may be more resistant to correction, requiring deeper shifts in worldview or moral framing to alter. Future research could explore this distinction more explicitly to inform strategies aimed at improving public understanding of economic inequality.

If BJW contributes to lower perceived inequality through heuristic or motivated processing, this has important implications for designing interventions aimed at improving public understanding of social disparities. Because BJW reflects a deeply rooted need to see the world as fair and predictable, it may resist direct challenge through factual information alone. However, some studies (Laurin et al., 2011)—and our own findings in this paper—suggest that BJW can be influenced, at least in the short term, through targeted messaging. In this paper, for example, we used an ostensible fairness index that presented participants with information portraying society as either fair or unfair. This manipulation led to short-term changes in BJW, indicating that contextual cues about fairness can shape individuals' belief in a just world, even temporarily. Such findings suggest that BJW is not entirely stable and can be responsive to how societal information is framed. Educational interventions that emphasize the structural roots of inequality, as well as messaging strategies that make systemic unfairness more salient, may therefore help counteract the perceptual biases associated with BJW. Framing inequality as a violation of core societal values—such as meritocracy or equal opportunity—might also reduce defensiveness and foster more accurate perceptions. Future research should explore which types of interventions are most effective in producing lasting changes in BJW and its impact on perceptions of inequality.

### 5.2 Conclusion

Inequality is arguably one of the most pressing social issues of our time and one root cause of a variety of social and individual ills (Brockhaus, 2020; Kondo et al., 2009; Obama, 2017; Wilkinson and Pickett, 2009) and yet, individuals often disagree on their evaluation of inequality or even the true extent of it (Gimpelson and Treisman, 2018; Hauser and Norton, 2017; Norton and Ariely, 2011). The present research demonstrates how subjective representations of inequality (in form of evaluations and size perceptions) are systematically related to individuals' just world beliefs. Given that recent research emphasizes the subjective component of financial inequality (Bobzien, 2020; Castillo et al., 2021; Gimpelson and Treisman, 2018; Oshio and Urakawa, 2014; Schneider, 2012; Willis et al., 2022), the present findings allow for a first look at what psychological variables affect subjective financial inequality. This perspective will contribute to a better understanding of how financial inequality influences individuals.

## Data Availability

The datasets presented in this study can be found in online repositories. The names of the repository/repositories and accession number(s) can be found below: https://osf.io/4ap63/?view_only=a807414c00524dbb9ebef1e50a7946b1.

## Footnotes

^1^Prior research suggested that participants sometimes do not understand graphical displays of inequality correctly (see Hauser and Norton, 2017; Norton and Ariely, 2011). The in-depth explanation and comprehension check were included to address this issue.

^2^Using the whole sample, without exclusions, attenuates the effects: The manipulation becomes less impactful *t*_(598)_ = 2.78, *p* =.003, *d* = 0.23, *95%CI* [0.07, 0.39], the effect on perceived inequality becomes insignificant *t*_(598)_ = −0.66, *p* =.255, *d* = −0.05, *95%CI* [−0.21, 0.11] and the effect on evaluated size becomes weaker but remains significant *t*_(598)_ = −2.43, *p* =.008, *d* = −0.20, *95%CI* [−0.36, −0.04].

^3^The questionnaires were part of a larger collaboration with other researchers which allowed for financing these two representative surveys. As a consequence, the questionnaire contained other variables that are not directly relevant to the present research question. The questionnaires were designed to address different research questions with a common focus on the general topic of financial inequality. One of the other research questions addressed the relation between subjective inequality and subjective well-being and whether this relation is moderated by BJW. Thus, the data from Study 2a (not 2b) were also part of two other studies. Importantly, these studies in no way addressed whether BJW affected subjective financial inequality.

^4^In Study 2b we used scales from 1 to 9 for reasons of consistency, because other variables in the questionnaire of Study 2b required this format.

^5^Including the whole samples in Study 2a and 2b did not change the results when predicting the perceived size of inequality (Study 2a: *b* = −0.12, *p* < 0.001, $\eta_{\text{p}}^{2}$ = 0.014; Study 2b: *b* = −0.13, *p* = 0.001, $\eta_{\text{p}}^{2}$ = 0.016), nor when predicting the evaluated size of inequality (Study 2a: *b* = −0.14, *p* < 0.001, $\eta_{\text{p}}^{2}$ = 0.019; Study 2b: *b* = −0.12, *p* < 0.001, $\eta_{\text{p}}^{2}$ = 0.014).

^6^The means and standard deviations of the variables could only be meaningfully interpreted within countries (e.g., income was measured in different currencies; years of schooling differed strongly between countries etc.) we therefore did not include them in the overview in Table 3.

^7^We also looked at the effects within countries. See Supplementary Table S1 for an overview of the results in the online supplement (https://osf.io/hkgw6?view_only=a807414c00524dbb9ebef1e50a7946b1).

^8^In Model 2 of Table 4 we included all interactions simultaneously. Including only religiosity or objective income inequality at a time does reveal both as significant moderators, suggesting that both may influence the effect of meritocratic beliefs on the two facets of subjective inequality via the same mechanism. Supplementary Table S2 displays the complete results of the individual moderations and control variables (https://osf.io/3u9as?view_only=a807414c00524dbb9ebef1e50a7946b1).
